# Evaluation of Hemostatic Aids in Laparoscopic Nephron-Sparing Surgery

**DOI:** 10.1100/tsw.2011.7

**Published:** 2011-01-18

**Authors:** Saleh Binsaleh

**Affiliations:** Department of Surgery, Faculty of Medicine, King Saud University, Riyadh, Saudi Arabia

**Keywords:** laparoscopy, partial nephrectomy, hemostasis, nephron-sparing surgery

## Abstract

Partial nephrectomy is considered the standard of care for the management of small renal masses, and laparoscopic techniques are becoming popular for multiple reasons, one of which is minimal invasiveness. On the other hand, kidneys are extremely vascular organs, and renal hemorrhage is a major cause of morbidity after laparoscopic partial nephrectomies. Control of bleeding and management of calyceal injuries can be difficult and make the procedure technically challenging. This review looks at the various energy sources and hemostatic agents that are available to reduce bleeding during laparoscopic partial nephrectomies.

## INTRODUCTION

One of the essential elements in partial nephrectomy is to achieve secure renal parenchymal hemostasis. Concerns regarding hemostasis have precluded widespread use of laparoscopic partial nephrectomy (LPN) for all patients who would be candidates for open partial nephrectomy[1,2,3].

In LPN, the most commonly and securely used technique for achieving hemostasis from the significant inter- and intralobar parenchymal vessels, which are transected during LPN, is precise suture ligation followed by a tight hemostatic reapproximation of the renal parenchyma (renorrhaphy) over absorbable bolsters, with the renal hilum cross-clamped, similar to open partial nephrectomy.

The use of various hemostatic techniques and agents has been reported widely in LPN series. This article provides an update on the recent publications and advances in the field of laparoscopic renal parenchymal hemostasis.

## HEMOSTATIC AIDS

Initial management of acute bleeding in laparoscopic surgery consists of a direct presser, which can be applied with a laparoscopic grasping instrument, a laparoscopic kitner, or the suction tip. To aid in maintaining compression, a rolled-up piece of methylcellulose or gauze can be utilized to compress the bleeding area. Laparoscopic suturing, clips, and vascular staplers are additional methods for obtaining vascular control. Use of the LapraTy® clip (Ethicon EndoSurgery; Somerville, NJ) may greatly facilitate the repair of bleeding vessels by reducing the time required for suture tying. Besides mechanical means of obtaining hemostasis, a number of compression and energy-based technologies have been utilized to achieve renal parenchymal hemostasis.

## Double-Loop Tourniquet

This device consists of two U-loop strips of knitted tape extending from a 17F plastic sheath. The device has been proposed to achieve regional vascular control by circumferential compression of the renal parenchyma during a polar partial nephrectomy. In describing their technique, Gill et al.[4] placed one double loop around the upper and one around the lower renal poles, cinching the loop around the pole containing the tumor and leaving the other one loose, thus securely entrapping the kidney and achieving a tourniquet effect. The renal artery is not occluded; hence, minimizing ischemic renal damage. Additional advantages include short warm ischemia time and maintaining good perfusion to the uninvolved pole. Although it is effective in the smaller kidney of the experimental porcine model, such renal parenchymal tourniquets are clinically unreliable in the larger human kidney, where persistent pulsatile arterial bleeding has been noted from the parenchymal cut edge despite tourniquet deployment. Additional practical problems include the potential for premature tourniquet slippage causing significant hemorrhage, renal parenchymal fracture owing to too tight cinching of the tourniquet, and the lack of applicability for tumors in the middle part of the kidney[4].

## Cable Tie

This is another tourniquet-like technique to control bleeding from the resection site. McDougall et al. first reported the use of a plastic cable tie for LPN in a pig model[5]. Then Cadeddu et al.[6] reported its use in the clinical setting, where the tumor was exposed and the cable tie was applied in a loose loop and positioned around the pole between the tumor and the renal hilum. The tie was then tightened to render the entire involved pole ischemic, then the tumor excised. Similar caveat can be made on the cable tie as with the double-loop tourniquet.

## The Argon Beam Coagulator

The argon beam coagulator conducts a radiofrequency current to the tissue along a jet of inert, nonflammable argon gas. Argon gas has a lower ionization potential than air and, consequently, directs the flow of the current. It may also blow away blood and other liquids on the tissue surface, enhancing visualization of the bleeding site as well as eliminating electric current dissipation in the blood. Smoke is reduced because the argon gas displaces oxygen and inhibits burning. One initial study to assess its efficacy in the clinical setting comes from Postema et al.[7] who studied the blood loss, the time needed to achieve adequate hemostasis, and histological findings after liver resection in 12 pigs using argon beam coagulation or suture ligation only, the mattress suture technique, and tissue glue application. Argon beam coagulation resulted in less tissue damage than tissue glue or mattress suturing, and it was concluded that the argon beam coagulator is an efficient device for achieving hemostasis following partial hepatectomy in the pig and causes only a moderate tissue reaction.

In the urologic literature, clinical data on human partial nephrectomy are lacking, although its benefit as a surface coagulator can be inferred from the other parenchymal efficacy studies.

The argon beam coagulator is obviously insufficient for controlling the pulsatile arterial hemorrhage from larger intrarenal vessels.

## Ultrasonic Shears

Ultrasonic shears (Harmonic Scalpel) are a form of energy that simultaneously divide and coagulate tissue using a titanium blade vibrating at 55,000 Hz. The resulting temperature (ranging from 50 to 100°C) causes denaturing protein coagulum. In LPN, this is used for tumor excision with or without vascular clamping. Harmon et al.[3] evaluated its use in 15 patients undergoing LPN with small tumors (mean size of 2.3 cm) without vascular clamping, and reported a mean blood loss of 368 ml and a mean operative time of 170 min. They concluded the safety of this device for parenchymal resection without vascular control. Guillonneau et al.[8] performed a nonrandomized retrospective comparison of two techniques for LPN, without and with clamping of the renal vessels. In group one (12 patients), partial nephrectomy was performed with ultrasonic shears and bipolar cautery without clamping the renal vessels. In group two (16 patients), the renal pedicle was clamped before tumor excision. Mean renal ischemia time was 27.3 min (range 15 to 47) in group two patients. Mean laparoscopic operating time was 179.1 min (range 90 to 390) in group one compared with 121.5 min (range 60 to 210) in group 2 ($p = 0.004$). Mean intraoperative blood loss was significantly higher in group one than in group two (708.3 vs. 270.3 ml, $p = 0.014$). Surgical margins were negative in all specimens.

Although they offer the advantage of tumor excision without vascular occlusion and hence reduce the possibility of renal ischemic damage, the disadvantage of ultrasonic shears includes tissue charring, which causes tissue to adhere to the device, creating an inexact line of parenchymal incision with poor visualization of the tumor bed. They are also inadequate as the sole hemostatic agent for controlling major renal parenchymal bleeding[9].

## Water (Hydro) Jet Dissection

Hydro-Jet technology utilizes an extremely thin, high-pressure stream of water. This technology has been routinely used in industry as a cutting tool for different materials, such as metal, ceramic, wood, and glass. Recently, Hydro-Jet technology has been used for dissection and resection during open and laparoscopic surgical procedures. A high-pressure jet of water forced through a small nozzle allows selective dissection and isolation of vital structures, such as blood vessels, collecting systems, and nerves.

Shekarriz et al. investigated this technology during LPN in the porcine model[10] and reported a virtually bloodless field, with the vessels and collecting system preserved. Moinzadeh et al.[11] evaluated the Hydro-Jet–assisted LPN without renal hilar vascular control in the survival calf model. Twenty kidneys were investigated and pelvicaliceal suture repair was found necessary in five of 10 chronic kidneys (50%). Mean Hydro-Jet partial nephrectomy time was 63 min (range 13 to 150), mean estimated blood loss was 174 ml (range 20 to 750), and mean volume of normal saline used for hydrodissection was 260 ml (mean 50 to 1,250). No animal had a urinary leak.

To date, no human studies for water-jet dissection in LPN have been described.

## Microwave Coagulation

A microwave tissue coagulator was introduced by Tabuse in 1979[12] for hepatic surgery and has subsequently been shown to coagulate vessels as large as 3–5 mm in diameter. This technique utilizes needle-type monopolar electrodes to apply microwave energy to the tissue surrounding the electrode. These microwaves comprise the 300- to 3000-MHz range of the electromagnetic spectrum and generate heat at the tip of the electrode, leading to formation of a conical-shaped wedge of the coagulated tissue.

In urology, microwave energy has been successful in prostate surgery for both benign enlargement and malignant disease. A microwave coagulator has been utilized clinically by Kagebayashi and colleagues and Naito and associates for open partial nephrectomy. Several other studies have reported the usefulness of this apparatus in open partial nephrectomy, especially in wedge resection of small renal tumors without renal pedicle clamping[13,14,15,16,17,48]. For LPN, Yoshimura et al.[18] reported its use in six patients with small exophytic renal masses (11–25 mm in diameter) without renal pedicle clamping at a setting of 2450 MHz. Mean operating time was 186 min (131 to 239) and blood loss was <50 ml. Complications were mild and tolerable, and there was no significant deterioration of renal function or urinary leak. Terai et al.[19] evaluated the same technique in 19 patients with small renal tumors, 11–45 mm in diameter, without hilar clamping. The mean operative time was 240 min with minimal blood loss in 14 patients, and 100–400 ml in four patients. In one patient, frozen sections revealed a positive surgical margin and additional resection was performed. Postoperative complications included extended urine leakage for 14 days, arteriovenous fistula, and almost total loss of renal function in one patient. With the median follow-up of 19 months, no patients showed local recurrence or distant metastasis by computed tomography (CT) scan. The authors stressed the fact that the indication of this procedure should be highly selective in order to minimize serious complications secondary to unexpected collateral thermal damage to surrounding structures.

## Radiofrequency Coagulation

Investigators have successfully used interstitial ablative technologies (like radiofrequency ablation and cryotherapy) as definitive *in situ* management of select renal lesions[20,21,22], but in this technique, as ablated tumors are left *in situ*, the effectiveness of ablation in the target lesion and the cost of radiographic follow-up have created postoperative concerns. Hence, radiofrequency-assisted LPN has emerged. Similar to microwave coagulation, radiofrequency (RF) coagulation can be used prior to partial nephrectomy to achieve energy-based tissue destruction followed by resection of the ablated tissue in a relatively bloodless field, without the need for hilar clamping.

In this technique, RF energy is applied by electrodes placed into a grounded patient to produce an electric current. Impedence within the tissue leads to heat production, which results in temperatures sufficient to cause tissue coagulation.

Gettman et al.[23] reported this technique in 10 patients with both exo- and endophytic tumors, 1.0–3.2 cm in diameter. The median operative time was 170 min and the median blood loss was 125 ml. This technique resulted in complete tissue coagulation within the treated volume, thereby facilitating intraoperative visualization, minimizing blood loss, and permitting rapid and controlled tumor resection. The renal architecture was preserved, allowing accurate diagnosis of renal cell carcinoma and angiomyolipoma in nine and one cases, respectively. No perioperative complications occurred.

More recently, Urena et al.[24] reported their experience with this technique in 10 patients, including nine with solid renal masses and one with a complex cyst. In all cases, the renal hilum was dissected and the renal vessels were isolated, but none had renal vascular clamping. Mean tumor size was 3.9 cm (2.1–8). The mass had a peripheral location in seven cases and a central location in three. Mean operative time was 232 min (144–280) and mean blood loss was 352 ml (20–1000). One patient received a blood transfusion and all tumor margins were negative. One patient had a short period of urine leakage from the lower pole calix, which was managed by ureteral stenting and Foley catheter drainage of the bladder.

Although this technique resulted in successful resection of exophytic and partial endophytic lesions in a relatively bloodless field without the need for vascular clamping, its applicability in central or deep lesions is still in question and longer follow-up for oncologic evaluation is still awaiting.

## Lasers

Several lasers have been developed specifically for surgical applications, used to cut or vaporize tissue while leaving a coagulated field. Their efficacy to coagulate or excise tissue is regulated by a specific wavelength, energy, or power setting and mode of operation (continuous or pulsed)[25].

Several kinds of laser energy have been tested for parenchymal transection during LPN[25,26,27]. The use of laser fibers with the specific application of tissue welding is based on delivering energy to the target lesion, with heat absorption resulting in thermocoagulation. This modality avoids needle trauma and suture reaction, and may allow shorter operative time and less bleeding, although it presents thermal damage in the tissue with indirect contact[28,29,30,31]. The search for the ideal hemostatic method still continues since no single laser technique was proven to have ideal results.

Studies on laser open partial nephrectomy using the $CO_2$, Nd:YAG, and holmium lasers have previously been published[26,32]. Since this early experience, several authors have reported the use of lasers for LPN in animal models, as well as in the clinical field.

Lotan et al.[32] studied the use of the holmium laser for partial nephrectomy in the porcine model. The authors performed transperitoneal lower pole LPN in five pigs. Fibrin glue was applied to the nephrectomy bed to seal the collecting system. All cases were performed with adequate hemostasis and without the need of further hemostatic devices.

Lotan et al.[33] described the first clinical report of laser during LPN using the holmium:YAG laser in three patients. There was minimal blood loss and no need for hilar clamping. Although the laser alone was hemostatic, the authors used fibrin glue in two cases and oxidized cellulose in one case to reinforce the tissue against delayed bleeding. There were no perioperative complications and the average hospitalization was 3 days. The advantages of the Ho:YAG laser are simplicity of use and relative low cost, the ability to cut and coagulate tissues with minimal damage to the adjacent renal parenchyma, and the preservation of as much normal tissue as possible. The disadvantages include the smoke created and the splashing of blood on the camera, particularly when transecting larger vessels. The authors concluded that with high power settings (0.2 J/pulse at 60 pulses/sec and 0.8 J/pulse at 40 pulses/sec), the Ho:YAG laser can be used as an effective hemostatic tool in LPN.

The use of the diode laser in LPN was reported by Ogan et al.[34]. They performed transperitoneal lower pole LPN in five pigs without the need for hilar occlusion using a 980-nm diode laser. The laser hemostasis was insufficient in three cases, requiring adjunctive measures, such as hemostatic clips, to stop bleeding. The mean operative time was 126 min, the mean blood loss was 150 ml (50–300 ml), and no cases of urine leak were observed at 2 weeks follow-up. The authors concluded that the diode laser is feasible on the porcine model and limited its use in humans to small peripheral tumors. The limitation of this laser was observed in controlling large vessels. Fibrin glue was applied to all partial nephrectomies, resulting in sealing of the collecting system in all cases, but it was not known if the sealing occurred as result of the glue or the laser.

The same group utilized an 810-nm pulsed diode laser (20 W) plus a 50% liquid albumin-indocyanine green solder in five pigs, demonstrating the tissue-welding qualities of lasers[35]. All surgeries were performed without complications with a mean operative time of 82 min, average blood loss of 43.5 ml, and mean warm ischemia time of 11.7 min. There was no evidence of urine leak or delayed hemorrhage in any of the animals. Histologic studies showed good preservation of renal parenchyma beneath the solder.

The main advantage of this soldering technique includes the ability to close the collecting system and control bleeding during LPN, with short warm ischemia time (<12 min). In this study, the laser was able to control large vessels, mimicking human LPN, and the violated collecting system was fixed with the solder without problems.

The KTP laser has been recently tested for LPN in the calf model[36]. Using six calves, the authors successfully completed the operation without hilar clamping in 11 out of 12 procedures. One animal required temporary occlusion of the hilum for hemorrhage not controlled with the laser. The histological analysis revealed minimal effect on the adjacent area to the excision. The unique feature of the KTP laser includes the 532-nm wavelength, with specific uptake by hemoglobin. The authors believed that this aspect yielded excellent hemostasis in the robust calf model. In addition, minimal blood splatter was noted, given decreased bleeding and thermomechanical ejection when compared to the Ho:YAG laser.

## Biologic Tissue Sealants

Biologic fibrin sealants are increasingly described in published studies for various surgical specialties[37], and in urology, these agents have been used during pyeloplasty, for ureteric repair, renal trauma, the treatment of urinary fistulae, and open and laparoscopic partial nephrectomy since 1979[38,39,40]. A recent survey of 193 members of the World Congress of Endourology revealed that 68% of surgeons routinely utilized fibrin sealant to assist with hemostasis during LPN[41]. Table 1 illustrates the hemostatic agents and tissue adhesives available in the U.S.

**TABLE 1**
**Hemostatic Agents and Tissue Adhesives Available in the U.S.**

| Brand Name (®) | Component | Manufacturer | Use |
|---|---|---|---|
| Tisseel VH, Crosseal | Fibrin sealant | Baxter | Hemostatic, tissue adhesive |
| FloSeal | Gelatin matrix thrombin | Baxter | Hemostatic |
| Thrombin-JMI | Thrombin | Jones Pharma | Hemostatic |
| Gelfoam | Gelatin sponge | Pharmacia & Upjohn | Hemostatic |
| Surgicel | Oxidized cellulose | Ethicon | Hemostatic |
| Actifoam | Collagen sponge | CR Bard | Hemostatic |
| Avitene | Collagen fleece | Davol | Hemostatic |
| NovoSeven | Recombinant factor VII a | Novo Nordisk A/S | Hemostatic |
| CoSeal | Polyethylene glycol | Baxter | Tissue adhesive |
| Dermabond | Cyanoacrylate | Ethicon | Tissue adhesive |
| BioGlue | Albumin glutaraldehyde | Cryolife | Hemostatic, tissue adhesive |

One example of these is the gelatin matrix thrombin sealant (FloSeal®, Baxter), approved by the Food and Drug Administration in 1999. This agent is composed of glutaraldehyde cross-linked fibers derived from bovine collagen. Its basic mechanism of action is to facilitate the last step of the clotting cascade, conversion of fibrinogen to fibrin. Furthermore, cross-linking of soluble fibrin monomers creates an insoluble fibrin clot that acts as a vessel sealant. Not dependent on the natural coagulation cascade for its efficacy, the gelatin granules (500–600 μm size) swell on contact with blood, creating a composite hemostatic plug with physical bulk that mechanically controls hemorrhage[42].

Richter et al.[43] and Bak et al.[44] described the use of gelatin matrix thrombin sealant in LPN. In the 16 cumulative patients in these two small series, no renal suturing was used. All tumors were somewhat superficial, with no patient undergoing pelvicaliceal repair. The median blood loss was 109 and 200 ml, respectively; no patient required blood transfusion; and none developed postoperative hemorrhage.

Another example of tissue sealant is Tisseel® fibrin sealant (Baxter), a complex human plasma derivative with significant hemostatic and tissue-sealant properties. This fibrin sealant includes a concentrated solution of human fibrinogen and aprotinin, which, on delivery, is mixed equally with a second component consisting of thrombin and calcium chloride. The addition of aprotinin helps to slow the natural fibrinolysis occurring at the resection site. With time, natural bioabsorption of the Tisseel will result from plasma-mediated lysis[45].

Bovine serum albumin and glutaraldehyde tissue adhesive (BioGlue®) is another example of these sealants that has been introduced recently to urologic surgery. Glutaraldehyde exposure causes the lysine molecules of the bovine serum albumin, extracellular matrix proteins, and cell surfaces to bind to each other, creating a strong covalent bond. The reaction is spontaneous and independent of the coagulation status of the patient. The glue begins to polymerize within 20–30 sec and reaches maximal strength in approximately 2 min, resulting in a strong implant. The degradation process takes approximately 2 years, and it is then replaced with fibrotic granulation tissue. Hidas et al.[46] studied the feasibility of using BioGlue to achieve hemostasis and prevent urine leakage during open partial nephrectomy in 174 patients. A total of 143 patients underwent the surgery with the traditional suturing technique (suture group) and 31 patients underwent a sutureless BioGlue sealing-only procedure (BioGlue group). The use of BioGlue reduced the mean warm ischemic time by 8.8 min (17.2 vs. 26 min, $p = 0.002$). The mean estimated blood loss was 45.1 ml in the BioGlue group and 111.7 ml in the suture group ($p = 0.001$). Blood transfusion was required in one patient (3.2%) of the BioGlue group and 24 (17%) in the suture group ($p = 0.014$). None of the patients treated with BioGlue developed urinary fistulae compared with three (2%) in the suture group.

The use of other local hemostatic agents, such as gelatin (Gelfoam®, Pharmacia & Upjohn), thrombin, oxidized regenerated cellulose (Surgicel®, Ethicon), and microfibrillar collagen (Avitene®, Davol), has been fraught with difficulties in application, particularly in parenchymal bleeding sites without a dry surface, in difficult-to-reach locations, and by a lack of efficacy in anticoagulated patients[43].

In renal surgery, only a few studies, none of them prospective and randomized, have tried to evaluate the efficacy of tissue sealants, fibrin sealant in particular[37,38,39,47]. The general observation from these studies is that a relatively dry parenchymal surface is essential before application of conventional fibrin sealants and if this can be achieved, minor venous oozing can be stopped. It is worth mentioning that a number of these investigations that addressed the effectiveness of fibrin sealant used one or more additional methods of hemostasis, such as suturing or argon beam coagulation.

The disadvantages of biologic sealant technology include, in addition to its cost, allergic reaction, potential transmission of prion diseases because of its bovine derivation, and the need to mix two components and/or sequentially apply them. The risk of viral transmission with gelatin matrix thrombin sealant appears to be remote. Because it is essentially a hemostatic agent, some may be ineffective for sealing collecting system entries[42].

Fibrin sealants offer an effective adjunct for hemostasis, reinforcement of urinary tract closure, and adhesion of tissue planes[40], but they should not be viewed as a replacement for conventional sound surgical judgment or technique.

In the future, it is likely that newer potent bioadhesives may have a stronger role in obtaining renal parenchymal hemostasis.

## CONCLUSION

Laparoscopic nephron-sparing surgery continues to be an attractive treatment option, particularly in the current setting of more frequent radiographic identification of incidental small renal masses. The current review outlines the different sealants and laparoscopic instruments available for achieving hemostasis of the renal parenchyma. The complexity of nephron-sparing surgery, coupled with the significant risk of hemorrhage from the renal parenchyma, makes the laparoscopic approach more challenging. Clearly, there is no gold-standard single agent or combination of products that can be applied to all cases. The decision as to which technologies to use and how to manage the hilum should be made based on the surgeon's experience and comfort level, as well as the complexity of the case. By no means does the use of hemostatic agents and instruments preclude a sound laparoscopic suturing technique. Indeed, a combined approach, utilizing manual suturing and hemostatic technology, may be the best strategy.
